# Unlocking the secrets: exploring the influence of the aryl hydrocarbon receptor and microbiome on cancer development

**DOI:** 10.1186/s11658-024-00538-0

**Published:** 2024-03-06

**Authors:** Menatallah Rayan, Tahseen S. Sayed, Ola J. Hussein, Lubna Therachiyil, Zaid H. Maayah, Cristina Maccalli, Shahab Uddin, Jochen H. M. Prehn, Hesham M. Korashy

**Affiliations:** 1 Department of Pharmaceutical Sciences, College of Pharmacy, QU Health, Qatar University, P. O. Box 2713, Doha, Qatar; 2 Department of Physiology and Medical Physics, Royal College of Surgeons in Ireland, Dublin 2, Ireland; 3 Translational Research Institute, Academic Health System, Hamad Medical Corporation, Doha, Qatar; 4 Research Branch, Sidra Medicine, Doha, Qatar; 5 Dermatology Institute, Academic Health System, Hamad Medical Corporation, Doha, Qatar; 6 RCSI Centre for Systems Medicine, Royal College of Surgeons in Ireland, Dublin 2, Ireland

**Keywords:** Microbiome, Aryl hydrocarbon receptor, Cancer, CYP1A1, Environmental pollutants

## Abstract

Gut microbiota regulates various aspects of human physiology by producing metabolites, metabolizing enzymes, and toxins. Many studies have linked microbiota with human health and altered microbiome configurations with the occurrence of several diseases, including cancer. Accumulating evidence suggests that the microbiome can influence the initiation and progression of several cancers. Moreover, some microbiotas of the gut and oral cavity have been reported to infect tumors, initiate metastasis, and promote the spread of cancer to distant organs, thereby influencing the clinical outcome of cancer patients. The gut microbiome has recently been reported to interact with environmental factors such as diet and exposure to environmental toxicants. Exposure to environmental pollutants such as polycyclic aromatic hydrocarbons (PAHs) induces a shift in the gut microbiome metabolic pathways, favoring a proinflammatory microenvironment. In addition, other studies have also correlated cancer incidence with exposure to PAHs. PAHs are known to induce organ carcinogenesis through activating a ligand-activated transcriptional factor termed the aryl hydrocarbon receptor (AhR), which metabolizes PAHs to highly reactive carcinogenic intermediates. However, the crosstalk between AhR and the microbiome in mediating carcinogenesis is poorly reviewed. This review aims to discuss the role of exposure to environmental pollutants and activation of AhR on microbiome-associated cancer progression and explore the underlying molecular mechanisms involved in cancer development.

## Introduction

The human body is a complex ecosystem hosting trillions of commensal microbes, collectively known as “microbiota” [1, 2]. These microbes represent over 5,000 species, including archaea, bacteria, protists, viruses, and fungi, among which bacteria are the most abundant [3, 4]. The human microbiome refers to the collective genetic makeup and byproducts of these microorganisms that inhabit the human body [3]. Approximately 90% of human cells are associated with microbiota, while only 10% are microbiome-free [5]. Commensal microbes reside in numerous sites within the human body, including the skin, oral cavity, and, most prominently, the gut [3, 6].

The mammalian gut is arguably one of the most advanced and complex communities of commensal microbes and presents the highest concentration of microorganisms in the human body [1, 2, 7]. The gut microbiome has been characterized as “the last undiscovered human organ” due to its significant impact on human health [8]. Gut microbiota was shown to play a profound role in regulating various aspects of human physiology by producing metabolites, metabolizing enzymes, and toxins [9]. Consequently, it helps protect the body from pathogenic invaders, promotes the development of the immune system, aids in food digestion and nutrient uptake, and may even influence mood and behavior [3, 6, 9]. Moreover, through their interaction with epithelial and stromal cells, gut microbiota regulates mucosal immune homeostasis, acts as a barrier, controls pathogen overgrowth, and maintains host–microbiota symbiosis [2, 10–12]. Therefore, dysbiosis, an alteration in the composition or function of the gut microbiome in response to environmental or host-related changes in a way such that the balance between beneficial microorganisms and pathogenic microorganisms is lost[13, 14], has been increasingly linked to the emergence of diverse pathological conditions such as obesity [15–17], diabetes [18–20], cardiovascular diseases [21], neurological disorders [22–24], and even psychological disorders [25, 26]. Furthermore, altered gut microbiota contributes to developing autoimmune diseases such as rheumatoid arthritis and systemic lupus erythematosus [27, 28]. Recently, a growing body of evidence has revealed a link between gut microbiota and cancer [2, 26].

The normal gut microbiota is dominated by four major phyla of bacteria: Bacteroidetes, Firmicutes, Proteobacteria, and Actinomycetes [2]. Firmicutes and Bacteroidetes account for more than 90% of the bacterial population in the gastrointestinal tract (GIT), while Actinobacteria and Proteobacteria are less abundant [29]. Nevertheless, each individual’s exact composition of gut microbiota is distinctive [3]. In 2010, researchers suggested that individuals could be characterized on the basis of the significant constituents of their gut microbiome to possess distinct “enterotypes” which are shaped by factors such as geographical area, diet pattern, genetic features, lifestyle, and environmental factors [30–33]. For example, transplanting fecal microbes from obese donors to germ-free mice induced higher weight gain than similar mice receiving fecal microbiota from lean donors [33]. These findings highlight the physiological importance of microbiota equilibrium, which appears to be unique for each individual [3, 34]. It has been suggested that once established, the gut microbiome’s composition remains relatively unchanged and resilient throughout adult life. However, factors such as bacterial infections, use of antibiotics, smoking, and disease states may still alter the human gut microbiome’s composition [2, 3, 30–33].

## Gut microbiome and cancer

Over the years, several studies have begun to direct focus on the study of gut microbiota and its implications on human health. Advancements in biomedical research have shed light on how disruptions in the gut microbiota could likely contribute to the etiology of disease pathogenesis [35]. Although initial studies were specific to enteric infections [36], over the past decade, microbiome research has rapidly shifted from pathology-related laboratory-based studies toward research that enables the enumeration of specific intestinal bacteria specimens, their genes, and metabolic products [37]. The National Institute of Health in the USA launched the Human Microbiome Project to establish a microbial genome database. This database provided insight into the diversity of microbial organisms, their genomic information, and their interaction across several organ systems, including the GIT, oral cavity, nares, and breast [38]. Extensive advancements in high-throughput techniques such as transcriptomics, sequencing, and metabolomics studies have further facilitated the understanding of the association between gut microbiota and a spectrum of immunological, neuro-psychiatric [39], and allergic disorders [40], atherosclerosis [41], obesity and diabetes [42], Parkinson’s disease [43], autism [44], central nervous system dysfunction [39], and malignancies [41, 45].

An accumulating body of evidence has shown that the microbiome can influence the development, progression, and therapeutic outcomes of cancer patients [4, 6, 8, 26, 46]. Particularly, alteration in the gut microbiota and infection of tumors with the microbiota of the gut and oral cavity have been linked to the initiation and progression of various types of tumors, including gastric cancer [47, 48], colorectal cancer (CRC) [46], and esophageal cancer [49]. Besides, it was found that the effects of gut microbiome extend beyond the GIT, where it can promote carcinogenesis in distant organs such as the liver [50, 51], pancreas [52, 53], breast [54], and brain [55]. Around 20% of the worldwide tumors’ burden is estimated to be triggered or modulated by microbes and their byproducts [9, 56]. Several studies utilizing metagenomic approaches revealed novel pathogens enriched in different types of tumors compared to surrounding tissues or healthy individuals, indicating that the microbiome is now recognized as a prospective hallmark of cancer [57].

While alterations in gut microbiome composition can promote carcinogenesis, they, on the other hand, may reduce the risk of cancer development. Evidence highlights the microbiota’s dual function in maintaining individuals’ overall well-being [3]. Host microbiomes were shown to possess several functions that maintain homeostasis and prevent cancer development, including reinforcing mucosal barrier [58], enhancing antitumor immune responses [59], suppressing inflammation [60], and reducing genotoxicity [61].

On the other hand, the crosstalk among certain microbial species and infection of tumors with bacteria was found to influence cancer pathology by acting on DNA stability, cancer immune responses, and microenvironment composition [62, 63]. Alterations in the gut microbiome increase the risk of GIT malignancies and promote carcinogenesis by inducing chronic inflammation, releasing mutagenic metabolites, and promoting cell proliferation [4, 46, 64]. For instance, the colonization of *Helicobacter pylori* stimulates immune responses and persistent inflammation, resulting in gastritis and ultimately leading to gastric cancer [65–67]. Further analysis revealed that several genes of *Helicobacter pylori* alter tissue homeostasis, leading to the accumulation of cytokines and activation of other cancer-causing signaling, such as the β-catenin signaling pathway [65, 68]. On the other hand, eradicating *Helicobacter pylori* reduced the risk of developing gastric cancer, suggesting that it plays a significant role in the early stages of gastric carcinogenesis [69]. Moreover, epigenetic alterations could mediate microbiome effects on carcinogenesis [4]. For example, *Helicobacter pylori*-induced murine gastric tumor was associated with hypomethylation and reduction of miR-490-3p [70].

A previous study also demonstrated a connection between the gut microbiome and esophageal cancer. In a fluorescence in situ hybridization study with a CY3-labeled *Fusobacterium nucleatum* (*Fn*)-specific probe, *Fn* was significantly more abundant in esophageal squamous cell carcinoma (ESCC) tissues than adjacent non-cancerous tissues. It appeared to correlate with the tumor stage, with *Fn* DNA levels significantly higher in advanced ESCC (Stage III–IV) than in early-stage ESCC (Stage I–II). The presence of *Fn* within the deep layers of the tissue indicated that this was not surface-level contamination from the oral cavity. BALB/C nude mice subcutaneously injected with KYSE-450 ESCC cells had significantly higher tumor volumes when the cells were pretreated with *Fn* compared with the negative control [71].

Similarly, the development of CRC has also been associated with specific types of microbes [46]. The gut microbiota signature analysis between patients with CRC and healthy individuals revealed a statistically significant difference between the two groups [72, 73]. For example, certain bacterial species such as *Bacteroides fragilis* (*Bf*), *Fn*, and *Porphyromonas asaccharolytica* (*Pa*) were found to be enriched in fecal metagenomic samples isolated from patients with CRC [74–76], suggesting that gut microbiome could serve as a noninvasive diagnostic marker for CRC. Additionally, even within the same patient, significant differences were found in microbiota community arrangements in tumor tissues as compared to surrounding normal tissues, in which *Fusobacterium* and *Lactococcus* were overrepresented, whereas *Pseudomonas* and *Escherichia*/*Shigella* were underrepresented [3, 77, 78].

Moreover, unique metagenomic and metabolomic shifts have been noticed in different stages of CRC, starting from polypoid adenomas and intramucosal carcinomas and moving to more advanced and metastatic lesions [79]. Notably, *Fn*’s relative abundance increased continuously with disease progression from intramucosal carcinomas to more advanced stages [79]. On the other hand, *Atopobium parvulum* and *Actinomyces ondontolyticus* were enriched only in multiple polypoid adenomas and intramucosal carcinomas. These findings were derived from multiomics data of a large cohort (*n* = 616) and suggested that microbiome and metabolome changes start from the early stages of CRC, highlighting its potential etiological and diagnostic relevance [79, 80]. Mechanistically, preclinical studies demonstrated that *Fn* promotes CRC cell proliferation in vitro and patient-derived CRC xenograft models by modulating the Wnt/β-catenin signaling pathway [81, 82]. Additionally, *Fn* can suppress immune surveillance by inhibiting the cytotoxic responses of tumor-infiltrating lymphocytes and natural killer cells through binding to the T-cell immunoreceptor with Ig and ITIM domains (TIGIT), an inhibitory immune checkpoint, protecting *Fn* and surrounding tumor cells from immune-mediated killing [83]. Moreover, additional preclinical studies have further established a direct causative relationship between the microbiome and the development of several types of cancer. For instance, feeding fecal samples from patients with CRC to healthy animals induced higher proinflammatory cytokines, altered immune responses, and initiated procarcinogenic signals and tumorigenesis in both germ-free and conventional mice models compared with feeding control fecal samples from healthy individuals [84].

Nevertheless, the exact underlying mechanisms have not been fully elucidated. Numerous studies have been undertaken to understand the mechanisms behind dysbiosis-mediated carcinogenesis and suggested several potential mechanisms. These mechanisms include the promotion of an inflammatory tumor microenvironment, epithelial–mesenchymal transition, production of reactive oxygen species (ROS), and suppression of tumor immune surveillance [3, 5, 85].

## Mechanisms through which the gut microbiome induces cancer

**Fig. 1:**
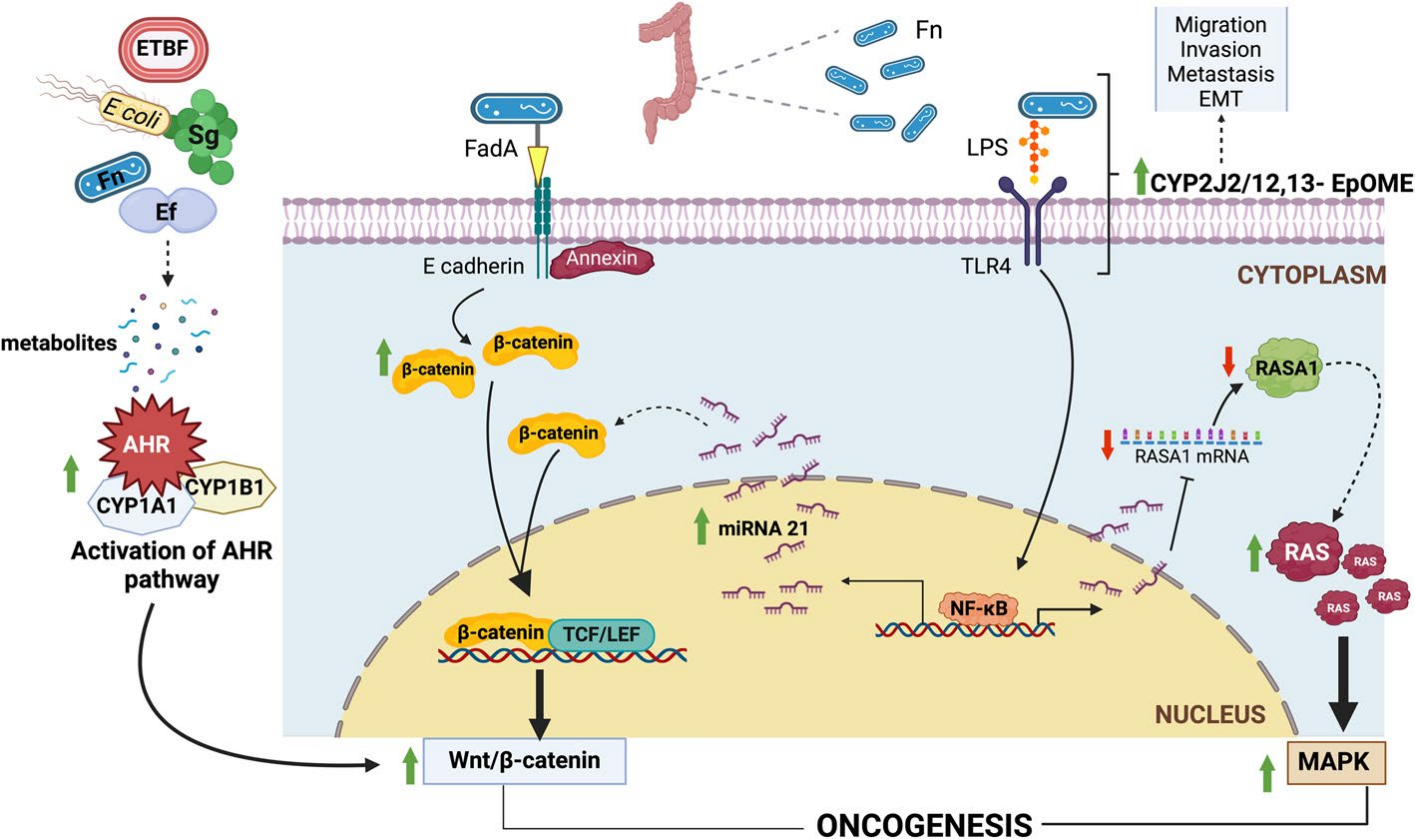
Mechanisms through which the gut microbiome contributes to oncogenesis. *Fn* expresses various virulent factors that activate prooncogenic pathways. FadA adhesin expressed by *Fn* binds to E-cadherin, leading to activation of Annexin 1 and activation of the Wnt/β-catenin signaling pathway. Activation of TLR4 by LPS activates NF-κB to upregulate the production of miRNA-21, which promotes oncogenesis by inducing the Wnt/β-catenin pathway and inhibiting RASA1, a GTPase activating protein that normally inhibits the Ras/Raf/MAPK pathway. Activation of TLR4 by LPS also upregulates the CYP2J2/12,13-EpOME pathway, promoting tumor migration, invasion, EMT, and metastasis. Microorganisms also secrete different metabolites which activate the AhR pathway, which contributes to oncogenesis through various mechanisms, one of which is through crosstalk with the Wnt/β-catenin signaling pathway. Created with BioRender.com

Gut dysbiosis alters the healthy gut microbiome’s composition and function, causing a loss of balance between beneficial and pathogenic microorganisms [13, 14]. Several intestinal microorganisms have been associated with carcinogenesis, including *Fn*, *Escherichia coli*, *Bf*, *Streptococcus gallolyticus* (*Sg*), *Clostridium septicum*, *Enterococcus faecalis (Ef)*, and *Pa* [14]. Several mechanisms have been proposed regarding how the gut microbiome can contribute to carcinogenesis, including but not limited to genotoxicity, immune tolerance, chronic inflammation, secreting oncogenic metabolites, expressing oncogenic virulence factors, and inducing oxidative stress [13]. Illustrative mechanisms are described in Fig. 1.

### Fusobacterium nucleatum (*Fn*)

Several studies have shown the role of *Fn* in gastrointestinal cancers and have highlighted several mechanisms through which *Fn* contributes to the development and progression of different cancer types. The effect of *Fn* on cancer cells appears to depend on the tumor type, as coincubation with *Fn* significantly stimulated the growth of several in vitro CRC cells, such as AA/C1/SB10C, HCT116, DLD1, SW480, and HT29 CRC cells [82, 86], but did not stimulate the growth of lung cancer (PC-9), prostate cancer (22RV1), bladder cancer (UMUC3), or breast cancer (MCF-7) cells [82]. Furthermore, upon assaying *Fn* levels in tissue sections from patients with colorectal adenomas and carcinomas, *Fn* levels were found to significantly increase with disease progression from tubulovillous adenoma to tubular adenoma, high-grade dysplasia, and eventually to CRC, and negatively correlate with overall survival [87]. Recently, *Fn* infection was reported to have the greatest negative prognostic impact in patients with high-risk, mesenchymal-rich tumors of the consensus molecular subtype 4 (CMS4). By contrast, this correlation is not observed in non-mesenchymal tumors (CMS1-3). Furthermore, the relative abundance of *Fn* in patients’ tumors was found to be positively associated with tumor proliferation, metastasis, and DNA damage [88]. In addition, several virulence factors expressed by *Fn* may contribute to these effects.

#### FadA interaction with Wnt/ β-catenin

*Fn* binds and invades AA/C1/SB/10C (aka 10C) CRC cells more efficiently than their non-cancerous counterpart AA/C1/SB (aka SB) cells. Annexin 1, a membrane protein previously found to be upregulated in 10C cells [89], seems to play an essential role in the interactions between *Fn* and cancer cells, as its downregulation in aka 10C cells reduced *Fn* binding and invasion. Conversely, overexpression of Annexin 1 in non-cancerous aka SB cells significantly increased *Fn* binding and invasion [82]. The growth stimulation appears to be mediated through FadA, as FadA-deficient *Fn* strains failed to stimulate CRC cell growth [82, 86]. FadA adhesin, a virulence factor expressed by *Fn* to mediate attachment and invasion, binds to E-cadherin (CDH1) and upregulates Annexin 1, which in turn activates Wnt/β-catenin signaling, leading to overexpression of oncogenes and inflammatory genes [82, 86].

#### Fap2 lectin

Fap2 lectin, another virulence factor expressed by *Fn*, recognizes and binds to d-galactose-β(1–3)-N- acetyl-d-galactosamine (Gal-Gal-NAc), which is overexpressed in CRC cells [90]. In another study, Fap2 was found to bind to the inhibitory TIGIT receptor on tumor-associated T cells and natural killer (NK) cells, leading to immune cells’ inhibition of cancer cell killing [91].

#### MicroRNA-21

Another mechanism through which *Fn* contributes to carcinogenesis is microRNA regulation. In HCT116 CRC cells, treatment with *Fn* followed by microarray analysis identified that *Fn* stimulates the Toll-like receptor 4 (TLR4) signaling to MYD88, which may be mediated through the binding of *Fn* lipopolysaccharide (LPS) to TLR4 [92], leading to activation of the nuclear factor κB (NF-κB). In turn, NF-κB upregulates the oncogenic miRNA-21 promoter activity, which reduces the levels of the RAS GTPase-activating protein (RASA1), a tumor suppressor that inactivates the oncoprotein RAS [93], a well-known activator of the Ras/Raf/MAPK pathway [94]. There could also be potential crosstalk between miRNA-21 and the Wnt/β-catenin pathway, as miRNA-21 knockdown in mice was found to significantly decrease tumor size and number, increase E-cadherin level, and decrease the expression of β-catenin, NF-κB, signal transducer and activator of transcription-3 (STAT3), Bcl-2, and SOX-9 [95].

#### CYP2J2 pathway

Activation of TLR4 by *Fn* could also contribute to carcinogenesis through upregulation of the cytochrome P450 (CYP) 2J2 (CYP2J2) and its metabolites, 12,13-epoxy octadecenoic acid (12,13-EpOME) production by activating the nuclear factor erythroid 2-related factor 2 (NRF2) pathway. Mechanistically, upon activation, NRF2 liberates from KEAP1 and then translocates to the nucleus and binds to the promoter region of the CYP2J2 to induce its transcriptional expression. A significant correlation was found between *Fn* level and CYP2J2 expression in human CRC tissues. Knocking down CYP2J2 in LoVo CRC cells significantly reduced the effect of *Fn* on promoting invasion, migration, epithelial–mesenchymal transition (EMT), and metastasis. These findings were validated in vivo using a model of azoxymethane-induced CRC in mice, where *Fn*-treated wild-type mice had significantly higher tumor loads than *Fn*-treated *Cyp2j5* knockout mice. This mechanism may contribute to the poor overall survival in patients with a high tumor expression of *CYP2J2*, as noted in The Cancer Genome Atlas (TCGA) database [96].

#### Autophagy and modulating chemoresistance

Coculture of the CRC cells, HCT116 and HT29, with *Fn* increased the expression of several autophagy-regulated markers, such as pAMPK, ULK1, pULK1, ATG7, and LC3-II. Correspondingly, the chemosensitivity of these cells to oxaliplatin and 5-fluorouracil, anticancer drugs, was reduced upon coculture with *Fn*, an effect that was abolished by treatment with chloroquine, a known autophagy inhibitor [97]. It has been demonstrated that *Fn* mediates autophagy and chemoresistance through downregulating miRNA-18a and miRNA-4802 expression. In patients with CRC, cancer recurrence was significantly correlated with high *Fn* levels, high expression of autophagy markers, and low expression of miRNA-18a and miRNA-4802 [97]. *Fn* was noted to also confer chemoresistance in ESCC cells through the activation of autophagy[98]. Oral treatment with the antibiotic metronidazole in mice with *Fn*-positive patient-derived CRC xenografts significantly reduced *Fn* load, tumor growth, and tumor cell proliferation[99].

#### Numb protein

Numb is a crucial cell fate regulator responsible for cell differentiation, tumor suppression, and rendering stemness onto cancer cells [100]. A recent study has shown that infecting CRC cells with *Fn* promotes cancer stem cell (CSC) features such as self-renewal through the activation of fatty acid oxidation and triglycerol synthesis. This leads to high lipid droplet production with Numb degradation. The study also showed that the promotion of CSCs by the action of the microbe is attributed to the activation of NF-κB, which led to the upregulation of carnitine palmitoyltransferase 1B, the enzyme that catalyzes the rate-limiting step of fatty acid oxidation, in CSCs [100]. Taken together, this study demonstrated that *Fn* renders stem cell-like features on CRC and could, therefore, promote cancer cells’ self-renewal ability.

### *Streptococcus gallolyticus* (*Sg*)

*Sg* is a strain of bacteria significantly associated with CRC. In vitro, the coincubation of CRC cells, such as HCT116, HT29, and LoVo, with *Sg* promoted their proliferation. However, this phenomenon was not observed in SW480 and SW116 CRC cells or normal human colon epithelial cells, indicating that the effect may be cell-specific. In an in vivo mouse model of azoxymethane-induced CRC, administration of *Sg* by oral gavage led to a significantly higher tumor burden compared with mice treated with normal saline [101]. This *Sg*-induced CRC proliferation in vitro and in vivo was mediated through the activation of β-catenin and its downstream targets c-Myc and cyclin D1, whereas the *Sg*-induced cell proliferation was abolished by knocking down β-catenin [101].

### *Bacteroides fragilis* (*Bf*)

Compared with nontoxigenic *Bf*, enterotoxigenic *Bf* (ETBF) was found to induce colon tumors in mice heterozygous for the adenomatous polyposis coli (*Apc*) gene. This effect is mediated through selective induction of STAT3, initiating a Th17-dominant immune response that contributes to carcinogenesis [102]. ETBF also produces an enterotoxin, known as *Bf* toxin (BFT), which, in addition to causing inflammatory diarrhea, it activates MAPK and NF-κB pathways [103, 104] and cleaves the extracellular domain of E-cadherin, causing the release of  β-cateninand and subsequent activation of downstream oncogenic pathways [105].

### *Escherichia coli*

Certain strains of *Escherichia coli*, namely the phylogenetic group B2, possess a conserved genetic island called “pks island” which codes for non-ribosomal peptide synthetases and polyketide synthetases. These enzymes produce a genotoxin, colibactin, that causes double-strand breaks in DNA and thus possibly contributing to CRC development [106]. These strains were significantly more prevalent in colon specimens from patients with CRC than in patients with diverticulosis as a non-cancer control group [107].

### *Enterococcus faecalis* (*Ef*)

*Ef* is a Gram-positive intestinal commensal bacterium that has been linked to CRC through two oxidative stress-mediated mechanisms. First, *Ef * produces extracellular superoxide, which causes lipid peroxidation leading to the formation of highly reactive electrophilic compounds, such as 4-hydroxy-2-nonenal. These compounds are potent genotoxins that bind with the DNA causing adducts and interfering with microtubule polymerization, thereby interfering with the formation of the mitotic spindle, causing tetraploidy and aneuploidy [108]. Second, *Ef* contributes to the risk of CRC by producing high levels of highly reactive hydroxyl radicals, causing oxidative stress [109].

### *Peptostreptococcus anaerobius* (*Pa*)

*Pa* was found to be more abundant in patients with CRC compared with healthy subjects. *Pa* promotes tumor cell proliferation by binding to TLR2 and TLR4 on colon cells to induce ROS production [110]. Furthermore, *Pa* binds to colon cell integrins using its putative cell wall binding repeat 2 (PCWBR2) receptor to activate phosphoinositide 3-kinases (PI3K)/Akt pathway, which stimulates cell proliferation and NF-κB-mediated inflammation [111].

## Environmental factors and microbiome-induced cancer

Interaction between the gut microbiome and environmental factors, such as diet, pollutants, and smoking, has recently been reported to contribute to cancers [112]. A strong positive association was found between *Fn*-positive colorectal tumors and consuming foods rich in red and processed meats [112]. It has been shown that the effect of exposure to environmental toxicants, such as aromatic hydrocarbons, may be dependent on the presence of specific gut microbiome populations [113]. For example, halogenated aromatic hydrocarbons (HAHs), such as 2,3,7,8-tetrachlorodibenzo-p-dioxin (TCDD), are persistent organic pollutants that bioaccumulate, particularly in fat tissue, through the food chain. As such, in addition to occupational exposure, human exposure to them is also possible through the consumption of meat, eggs, dairy products, and fatty fish [114]. Furthermore, polycyclic aromatic hydrocarbons (PAHs), such as benzo[a]pyrene (BaP), which have been linked to CRC in humans [115], are commonly found in processed meats and have been found to significantly upregulate oncogenes (e.g., KRAS) and downregulate tumor suppressor genes, such as TP53 [116]. In addition to their connection to cancer, exposure to PAHs was also found to induce a shift in the gut microbiome metabolic pathways, favoring a proinflammatory microenvironment [117]. It is well documented that PAHs and HAHs exert their toxicity and carcinogenicity through the interaction with and activation of a transcription factor, the aryl hydrocarbon receptor (AhR) [118]. However, the role of the AhR pathway in microbiome-mediated cancer remains unlocked. The next section of the review will discuss the crosstalk between AhR and microbiome in cancer development and progression.

### The aryl hydrocarbon receptor (AhR) pathway and cancer development

AhR is a cytosolic ligand-activated transcription factor that belongs to the bHLH-PAS (basic helix-loop-helix-Per/Arnt/Sim) protein family [119]. AhR is actively involved in the regulation of several transcription genes involved in cell proliferation [120], apoptosis [121], development, and immunomodulation [122]. Over the years, several studies have demonstrated activation of AhR plays a significant role in cancer initiation and promotion. AhR is activated by PAHs, such as BaP, benzoflavones, and benzanthracenes, and HAHs, such as dibenzofurans, biphenyls, and dioxins, the most notable of which is TCDD, the most potent AhR activator [118, 120]. Following ligand binding, AhR undergoes conformational changes and translocates to the nucleus, dimerizing with AhR nuclear translocator (ARNT) [121]. The formed AhR–ARNT complex then binds to specific DNA-responsive elements, the dioxin-responsive elements (DRE), to initiate the transcription expression of the AhR target genes, cytochrome P450 1A1 (CYP1A1) and CYP1B1 [122, 123]. The CYP1A1 and CYP1B1 then bioactivate pollutants into highly reactive and carcinogenic intermediates that initiate cancer [118, 124].

In support of the role of AhR in cancer development, researchers have identified upregulated expression of AhR in human and rodent tumors, such as breast cancer [125–127], lung adenocarcinoma [128, 129], and ovarian cancer [130], implicating the role of AhR in tumor progression. Interestingly, AhR also holds a functional role in the molecular mechanisms governing the development of CSCs, which are well-known tumor-initiating cells, along with being targets for several chemical carcinogens. AhR has not only been found to be constitutively expressed in cancer tissues but its activation has also been strongly linked to CSC progression and renewal [131]. Of particular interest, there was a study that explored the mechanistic role of the AhR/CYP1A1 signaling pathway in controlling the progression of breast CSCs. Findings from the study provide significant evidence that activation of the AhR signaling pathway leads to the development and progression of CSCs through inhibiting the tumor suppressor protein PTEN and the activation of the Akt and Wnt/β-Catenin pathways [132].

### AhR and microbiome crosstalk in cancer

Several recent studies, summarized in Table 1, have demonstrated a connection between AhR and the prooncogenic bacteria commonly found colonizing the tumor microenvironment. Analysis of the datasets from the TCGA database and the European Genome-phenome Archive (EGA) identified a significant association between AhR signaling and the abundance of *Fn* in CRC tissues [133]. Coculture of ESCC cell lines, such as KYSE-450 and KYSE-150, with *Fn* significantly promoted cell proliferation, colony formation, and cell migration compared to the control [71]. Interestingly, a subsequent high-throughput sequencing study on *Fn*-treated ESCC cells has identified CYP1A1, the major target gene for AhR pathway activation, as the most significantly upregulated gene. Similarly, patient-derived ESCC tissues with a high abundance of *Fn* had higher CYP1A1 protein levels than those with low *Fn*. In addition, inhibition of CYP1A1 either genetically using the shRNA lentivirus vector or pharmacologically using CH223191 abolished the effects of *Fn* on cell proliferation and colony formation in vitro and tumor growth in vivo [71]. In CRC cells, coculture of T18 cells with *Fn* was found to upregulate AhR, cancer stemness markers, CYP1A1, Wnt signaling, and MAPK signaling [133]. Similar findings were observed in another study, in which incubation of *Sg* with CRC cell lines, such as HT29, Caco-2, SW480, and HCT116, increased the expression of CYP1A1 and the CSC marker, aldehyde dehydrogenase 1 (ALDH1) in an AhR-dependent manner [134]. Furthermore, pretreatment of HT29 cells with *Sg* increased the genotoxicity of 3-methylcholanthrene, a PAH and CYP1A1 inducer [134]. These findings highlight the importance of the interaction between environmental toxins and the gut microbiome, as well as the role of the AhR/CYP1A pathway; however, more research is needed to determine whether the effect is due to CYP1A1- and/or AhR-dependent mechanisms.

**Table 1:** Summary of studies showing the effect of the microbiome on the AhR/CYP1 pathway and possible mechanisms involved

Model	Microorganism/metabolite	Effect on AhR	Effect on cancer	Mechanism	REF
THP-1 derived macrophages	*Fn*/kynurenine	↑Kyn (endogenous AhR agonist)	Immune tolerance	IDO activation by LPS-mediated release of TNF-⍺ and IL-6 leads to ↑Kyn	[141]
Administration of ampicillin to B6 pancreatic tumor-bearing mice	Bacterial indoles	↓cyp1a1 mRNA, ↓cyp1b1 mRNA, ↓cyp1a2 mRNA	↑PD-L1, ↑MHCII ↑IFNγ^+^TNFα^+^CD8^+^ T cells ↓Immune tolerance	AhR activation in tumor-associated macrophages by bacterial indoles suppresses CD8^+^ T-cell activity in the tumor microenvironment	[142]
*Lyz2*^*cre/*+^*Ahr*^*fl/fl*^ B6 pancreatic tumor-bearing mice					
TCGA PanCancer Atlas Studies (*n* = 10,071)		↑AhR with ↓survival	Enrichment of indole-producing bacteria ↓survival		
Caco-2 human CRC cells treated with butyrate ± TCDD	Butyrate (SCFA)	↑AhR mRNA, ↑CYP1A1 mRNA, ↑CYP1B1 mRNA ↑TCDD effect	Not measured	Butyrate acts as an HDAC inhibitor, increasing AhR recruitment to the CYP1A1 gene promoter	[143]
HepG2 treated with different SCFAs	SCFAs (butyrate, acetate, and propionate)	↑AhR activity, ↑CYP1A1 mRNA, ↑AhRR mRNA	Not measured	SCFAs act as HDAC inhibitors, increasing AhR recruitment to the cyp1a1 gene promoter	[144]
T18 CRC cells treated with *Fn* or formate	*Fn*/formate	↑AhR mRNA ↑ARNT, ↑CYP1A1 mRNA,	↑CSC markers (ALDH, CD44, OCT4, SOX2, CD133, CD24) ↑invasion, ↑metastasis ↑Wnt activation	*Fn*-derived formate activates AhR and Wnt signaling	[133]

## Mechanisms of AhR and microbiome crosstalk in cancer

**Fig. 2:**
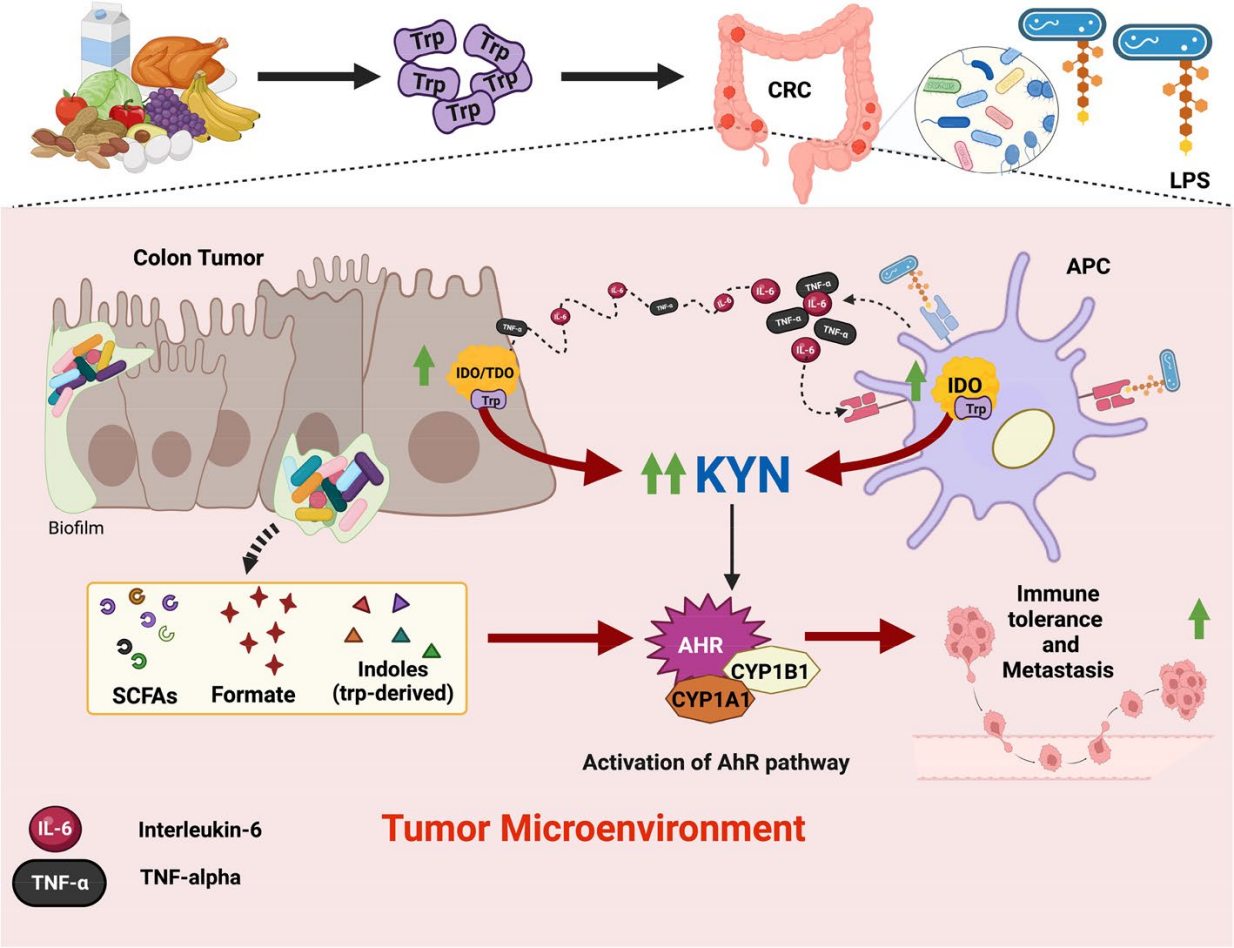
Mechanisms of AhR activation by microbiome in the tumor microenvironment. The microbiome within the tumor microenvironment secretes a variety of metabolites, including SCFAs, formate, and tryptophan-derive indoles, which promote immune tolerance and metastasis through activation of the AhR pathway. Furthermore, the release of TNF-⍺ and IL-6 in response to LPS in tumor-associated macrophages and dendritic cells (antigen-presenting cells; APCs) upregulates IDO activity in the resident APCs and tumor cells, leading to local overproduction of Kyn from tryptophan, which activates AhR to further promote immune tolerance. Created with BioRender.com

The main proposed mechanisms through which the gut microbiome activates AhR within the tumor microenvironment are illustrated in Fig. 2. Several studies have shown that the gut microbiome represents a rich source of AhR ligands, many of which are products of tryptophan metabolism [135]. Tryptophan is an essential amino acid obtained upon proteolysis of dietary protein. While most ingested tryptophan is absorbed into systemic circulation for use by the host in protein synthesis, approximately 10–20% of it undergoes further metabolism in the GIT by either host enzymes or gut microbiota [135]. There are four known competing catabolic pathways by which tryptophan can enter the GIT: kynurenine (Kyn) pathway (90–95%), serotonin pathway (2%), bacterial indoles pathway (5%), and tryptamine pathway (1%) [136].

### Kynurenine (Kyn)

One of the proposed theories regarding how the gut microbiome activates AhR to promote carcinogenesis is through the upregulation of the Kyn pathway. The Kyn pathway is a metabolic pathway that produces nicotinamide adenine dinucleotide (NAD) and is responsible for approximately 95% of total tryptophan catabolism [136, 137]. Kyn is synthesized mainly in the liver and brain by the enzyme tryptophan 2,3-dioxygenase (TDO) and by indoleamine 2,3-dioxygenase (IDO) in many tissues in inflammatory stimuli. TDO and IDO are overexpressed by tumor cells as have immune and neuroactive functions, in addition to the production of NAD+ and ATP in host cells [136, 137]. It has been reported that tumor-associated macrophages and dendritic cells (DCs) express high levels of IDO1, leading to local depletion of tryptophan and Kyn overproduction. Kyn is a weak AhR agonist and at high concentrations constitutively activates AhR to boost regulatory T-cell activity, leading to suppressing the activities of T helper cells (Th1, Th2), NK, and DCs. Such effects promote self-tolerance, immune evasion, and subsequently tumor survival [120, 136–139]. An in vivo study has demonstrated that administration of LPS upregulated TDO2 and IDO in mice, which was associated with increased serum Kyn and AhR activation [140], suggesting that carcinogenic microbes could activate AhR through a similar mechanism. Furthermore, infection of THP-1-derived macrophages with live/heat-killed *Fn* induced IDO expression in a time- and dose-dependent manner through LPS-mediated induction of the tumor necrosis factor (TNF-α) and IL-6, which are both IDO inducers [141].

### Bacterial indoles

The bacterial indole pathway in tryptophan catabolism may also play a role in activating AhR to induce cancer. This is evidenced by the observations that in pancreatic ductal adenocarcinoma patients, activation of AhR was found to positively correlate with the enrichment of indole-producing bacteria, while negatively correlates with overall survival [142]. Mechanistically, activation of the AhR in tumor-associated macrophages by tryptophan-derived bacterial indoles alters the cells polarization toward a protumor alternative antiinflammatory M2 phenotype and suppresses antitumor immunity. In this context, it has been shown that removal of dietary tryptophan, which cause AhR inhibition, and administration of ampicillin, an antibiotic that eradicates indole-producing Lactobacilli, all restored cytotoxic IFNγ + TNF⍺ + CD8 + T-cell function, increased tumor-associated macrophages expression of PD-L1 and MHC class II molecules, and reduced the expression of *Cyp1a1*, *Cyp1b1*, and *Cyp1a2* genes [142]. This reduced tumor growth and improved the efficacy of immune checkpoint blockade by PD-L1 inhibition. Importantly, knockdown of AhR in mice eliminated the effect of ampicillin on tumor growth, suggesting an AhR-mediated pathway [142]. Nevertheless, given the wide variety and bioactivity of indole compounds produced by different bacterial species, further studies are required to identify which specific indoles are produced by cancer-associated bacteria and could contribute to carcinogenesis.

### Short-chain fatty acids (SCFA)

Short-chain fatty acids (SCFAs), such as butyrate, acetate, and propionate, are produced from dietary fiber fermentation by microbiota and act as histone deacetylase (HDAC) inhibitors. HDAC inhibition increases the recruitment of AhR to its target gene CYP1A1’ promoter through the reshaping of chromatin and enhancing the expression of AhR-responsive genes CYP1A1, CYP1B1, AhR repressor in Caco-2 CRC cells and YAMC mouse colonic epithelial cells [143, 144]. Interestingly, in addition to enhancing the basal expression of AhR and its downstream genes, butyrate also synergistically enhanced TCDD-induced AhR activation [143], further highlighting the potential role of crosstalk between the gut microbiome and environmental toxins in carcinogenesis. Butyrate also enhanced AhR activation by microbiota-derived tryptophan metabolites, including indoles. Hence, rather than directly acting as AhR ligands, SCFAs synergize with bacterial-derived AhR agonists to facilitate the binding of the activated AhR complex to its target genes [143].

### Formate

Formate is a bacterial fermentation product that is known to play a role in cancer, but its role in the crosstalk between gut microbiome and AhR is still unexplored. Coculture with T18 CRC cells increases the *Fn* secretion of fermentation products, such as acetate, propionate, butyrate, and formate four-fold, with formate being the predominant fermentation product across different *Fn* strains [133]. Interestingly, AhR was the most significantly activated pathway following formate treatment. Incubation with *Fn* and formate-induced AhR nuclear translocation increased the invasion and migration of CRC cells, upregulated cancer stemness markers (ALDH1A1, CD44, OCT4, SOX2, CD133, and CD24), enhanced metastatic dissemination, and induced Wnt signaling. These effects were all reversed by AhR pharmacological inhibition by CH223191 [133]. However, the exact mechanisms through which formate activates AhR were not investigated, and further studies are required.

### Paradoxical modulation of AhR by microbiome and its effect on cancer (MR)

In the absence of cancer, AhR activation by the healthy gut microbiome has an important regulatory role in maintaining intestinal homeostasis and gut barrier integrity [145]. Patients with celiac disease have reduced levels of endogenous AhR ligands and consequently less intestinal AhR activity [146], and AhR knockout mice were found to be more susceptible to dextran sulfate sodium -induced colitis. Conversely, supplementation with β-napthoflavone, an AhR agonist, accelerated recovery from colitis [147]. These protective effects may be due to the AhR-mediated release of IL-10 and IL-22, which promote mucosal healing and inflammation resolution in inflammatory bowel disease [148–150].

The consequences of AhR activation hence appear to be ligand-specific, as while some AhR ligands that possess short half-lives and bind to AhR only transiently have been reported to be protective against colitis, other ligands have been reported to induce a proinflammatory response. The toxicities observed with carcinogens such as TCDD may hence be attributed to the persistent and potent occupation of the AhR [137] or to targeting different binding sites on the receptor [151, 152].

## Conclusions and remarks

In the last decade, valuable insights have been poured into identifying the role and involvement of the gut microbiome in modulating human physiology and disease. Due to the increased occurrence and higher mortality rate of cancer, the crosstalk between gut microbiota and cancer incidence has been well studied. Though reports have successfully proven their role in promoting tumorigenesis via altering the physiology and microenvironment and favoring tumorigenic environment, cancer development, progression, and altering treatment responsiveness, the role of other factors remains unexplored. As a significant pathway mediating gut homeostasis and as a sensor for several microbial metabolites, AhR could have a role in microbe-mediated tumorigenesis. Although studies are limited in this context, this could be a better endpoint for cancer maintenance and therapy. More studies identifying and testing the level of microbial metabolites activating AhR and their potential involvement in mediating cancer would shed new insights into managing cancer in a targeted therapy scenario. Furthermore, since most studies have focused on *Fn*, further research is required on the possible crosstalk between AhR and other bacterial species implicated in CRC, such as *Sg*, *ETBF*, *Escherichia coli B2*, *Enterococcus faecalis*, and *Peptostreptococcus anaerobius.* Additionally, the role of the microbiome in promoting immune responses and responsiveness to immunotherapy and the relevance of possible future investigations in light of the role of AhR need further investigation.

In this review, we attempted to highlight recent developments that point to a possible crosstalk between the AhR pathway and the microbiome in cancer initiation, promotion, and progression. Furthermore, revealing the mechanisms governing this crosstalk will provide future insights into disease understanding and pave the way for the revolutionization of targeted pharmaceuticals and therapeutics.

## Data Availability

Not applicable.

## Abbreviations

Apc: Adenomatous polyposis coli
AhR: Aryl hydrocarbon receptor
BaP: Benzo[a]pyrene
BFT: Fragilis toxin
CRC: Colorectal cancer
CSCs: Cancer stem cells
CYP: Cytochrome P450 proteins
CYP1A1: Cytochrome P450 1A1
CYP1B1: Cytochrome P450 1B1
CYP2J2: Cytochrome P450 2J2
DRE: Dioxin-responsive elements
EMT: Epithelial–mesenchymal transition
ETBF: Enterotoxigenic *Bacteroides fragilis*
Fn: *Fusobacterium nucleatum*
HDAC: Histone deacetylase
Kyn: Kynurenine
LPS: Lipopolysaccharide
NRF2: Nuclear factor erythroid 2-related factor 2
NF-κB: Nuclear factor κB
PDAC: Pancreatic ductal adenocarcinoma
Sg: *Streptococcus gallolyticus*
Stat3: Signal transducer and activator of transcription-3
TCDD: 2,3,7,8-tetrachlorodibenzo-p-dioxin
TDO: Tryptophan 2,3-dioxygenase
TIGIT: T cell immunoglobulin, and ITIM domain
TLR4: Toll-like receptor 4
